# Prechemotherapy Transperitoneal Robotic-Assisted Partial Nephrectomy (RAPN) for a Wilms Tumor: Surgical and Oncological Outcomes in a Four-Year-Old Patient

**DOI:** 10.3390/pediatric15030051

**Published:** 2023-09-21

**Authors:** Marcello Della Corte, Elisa Cerchia, Marco Oderda, Paola Quarello, Franca Fagioli, Paolo Gontero, Simona Gerocarni Nappo

**Affiliations:** 1 Division of Urology, Department of Oncology, School of Medicine, San Luigi Gonzaga Hospital, University of Turin, Regione Gonzole 10, 10043 Orbassano, Italy; 2 Division of Pediatric Urology, Regina Margherita Hospital, 10126 Turin, Italy; 3 Department of Urology, Città della Salute e della Scienza, Molinette University Hospital, Corso Bramante 88, 10126 Turin, Italy; 4 Division of Onco-Hematology, Department of Pediatrics and Pediatric Specialties, Regina Margherita Children Hospital, 10126 Turin, Italy

**Keywords:** Wilms tumor, nephron-sparing surgery, robotic surgery, partial nephrectomy, 3D reconstruction, DaVinci, metaverse, 3D virtual models

## Abstract

Background: Wilms tumor (WT) is the most frequent renal tumor in children. The SIOP-UMBRELLA Guidelines allow for nephron-sparing surgery (NSS) in syndromic patients, as well as in cases of small (<300 mL) non-syndromic unilateral WTs, without lymph node involvement, and with a substantial expected remnant renal function, following neoadjuvant chemotherapy. We present a case of prechemotherapy transperitoneal robot-assisted partial nephrectomy (RAPN) for a unilateral, non-syndromic Wilms tumor. Methods: A four-year-old child presented with a solid mass measuring 3.6 cm in diameter involving the upper right renal pole, incidentally detected during an abdominal echotomography. CT scan and abdominal MRI revealed no local infiltration or lymph node involvement, suggesting that the exophytic mass could be easily resected via an NSS robotic approach. Preoperative imaging did not strongly suggest WT. A virtual 3D reconstruction of the tumor was performed. Results: After the oncologic board approval, a robot-assisted partial nephrectomy with an intraperitoneal approach was performed. Histopathological analysis confirmed the diagnosis of WT. The patient subsequently received 10 doses of vincristine as adjuvant chemotherapy. A 28-month follow-up showed no tumor recurrence. Conclusions: Intraperitoneal RAPN may be an option for selected WT and warrants consideration as a challenging but advantageous approach.

## 1. Introduction

Wilms tumor (WT; nephroblastoma) is the second most common solid tumor and the most prevalent renal tumor in children [1]. It typically occurs in early childhood, with a peak incidence before the age of 5 years.

WT mostly presents as a painless mass in the upper abdominal quadrant, while hematuria is observed only in fewer than 20% of cases [2]. Diagnosis is typically made at abdominal ultrasound, which reveals a solid mass with smooth margins and may exhibit features such as vascular invasion, necrotic (hypoechoic), cystic (anechoic), and/or hemorrhagic/calcified/fatty (hyperechoic) areas [3]. Computed tomography (CT) during the venous phase provides an in-depth characterization of WT, which appears as a heterogeneous soft-tissue density mass, occasionally displaying calcified or fatty regions [4,5]. In younger children, magnetic resonance imaging (MRI) is considered the gold standard for staging due to its lack of ionizing radiation. WT exhibits heterogeneity on all MRI sequences, with varying blood content. It appears hypointense in T1 sequences, hyperintense in T2 sequences, and displays heterogeneous enhancement following gadolinium contrast media administration [2,4,6].

When dealing with suspected WT, it is important to fully evaluate differential diagnosis with neuroblastoma for the following reasons: (1) neuroblastoma commonly presents calcifications, a feature less frequently seen in WT; (2) neuroblastoma tends to encase vascular structures without invading them, whereas WT may extend into the renal vein and inferior vena cava; (3) neuroblastoma is not typically well circumscribed, as it often exhibits more diffuse or infiltrative growth patterns; (4) neuroblastoma frequently crosses the midline, extending beyond its original location, and can involve the diaphragm and the spinal canal; (5) neuroblastoma is often associated with retroperitoneal lymph node involvement, a characteristic less commonly observed in WT [7].

Additionally, there are less frequent differential diagnoses to consider: (1) cystic partially differentiated nephroblastoma, a condition that presents as a multicystic unilateral lesion, usually without solid components; (2) renal rhabdoid tumors, often displaying tumor lobules mixed with necrotic or hemorrhagic areas, subcapsular fluid collections, more calcifications than WTs, and a higher tendency to invade nearby vessels or structures; (3) clear cell sarcoma, which can be distinguished from other tumors when concurrent skeletal metastases are present; and (4) childhood renal cell carcinoma, which is extremely rare and appears as a solid mass with cystic areas or calcifications, and may show contrast media uptake.

In very rare cases, WT can be confused with benign conditions, including pediatric cystic nephroma (typically lacking solid nodular components) [8], renal abscess, hydronephrosis, and angiomyolipoma [2,3,4,5,6,7].

Once the diagnosis of WT has been established, surgery is the crucial step. The Children’s Oncology Group (COG) and the International Society of Pediatric Oncology (SIOP) differ on the surgical timing: the first recommends adjuvant treatment while the second dictates neoadjuvant chemotherapy [9]. Both COG and SIOP require radical nephrectomy together with lymph node sampling for staging purposes.

Different from renal tumors in adults, nephron-sparing surgery (NSS) is not recommended in children, unless in very selected cases. COG guidelines accept NSS in patients with a solitary kidney or a horseshoe kidney and in patients with genetic syndromes prone to oncological recurrences (i.e., bilateral WT, mutation). SIOP guidelines also permit NSS for non-syndromic unilateral WT with a small volume (<300 mL) with an expected average remaining kidney function and when lymph nodes have not been involved [10], although this practice is not evidence-based and must be performed only in selected centers with a high volume of cases and under established protocols [9].

Over the last 30 years, minimally invasive surgery (MIS) has seen renewed use in pediatric and urology surgery, from standard laparoscopic to robotic techniques, but only recently has MIS gained ground in pediatric urologic oncology with a few described applications to renal tumors [11].

In particular, a few cases of RAPN for WT have been described, all limited to syndromic patients prone to recurrent malignancies [12,13,14].

The aim of this paper is to present a case of a 4-year-old child treated with prechemotherapy RAPN for a unilateral WT.

## 2. Case Report

A four-year-old girl was admitted to our emergency department with a fever, abdominal pain, and diarrhea persisting for two days, which responded to acetaminophen.

The patient’s family and clinical history were uneventful.

Upon physical examination, the child was in good general condition, weighing 14 kg and measuring 107 cm in height. The abdomen was soft and painless, with no palpable masses, and all other findings were normal. She had fever, with a body temperature of 38.5 °C, oxygen blood saturation of 98% (in ambient air), no signs of dyspnea, and regular heart rate.

Urine and blood tests came back normal, except for a C-reactive protein level of 200 mg/L (normal range: 8–10 mg/L). Thoracic ultrasonography (US) revealed scattered B-lines and an irregular bilateral basal pleural line. The left lung showed signs of consolidation with an associated 7.7 mm × 10 mm (posterior × axillary) pleural effusion.

**Figure 1:**
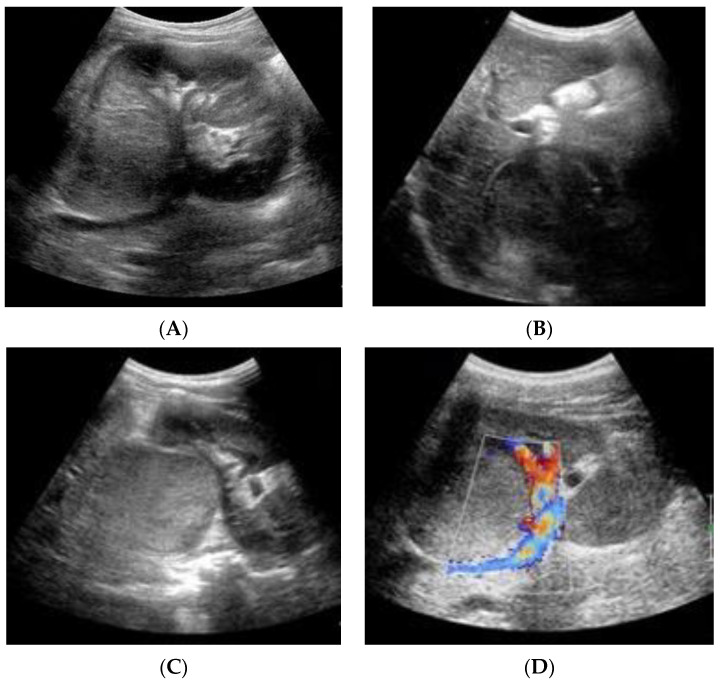
(**A**–**D**). Abdominal US: (**A**) A solid, roundish mass is visible at the upper pole of the right kidney. (**B**) The mass exhibits homogeneous isoechoic content, with an easily distinguishable pseudocapsule. No signs of involvement with neighboring organs are recognized. (**C**) Transverse scan of the tumor. (**D**) The color Doppler shows no evidence of renal vein thrombosis.

Abdominal US displayed an echogenic solid roundish mass of 3.6 cm diameter in the hepatorenal space. The mass was localized at the upper right renal pole, partially exophytic, with a hypoechoic homogeneous structure and a claw sign; no renal vein involvement was seen and the remaining organs were unharmed (Figure 1).

A broad-spectrum antibiotic therapy was initiated (parenteral ceftriaxone and oral clarithromycin), leading to a rapid improvement in body temperature, relief of abdominal pain, and normalization of blood test results.

**Figure 2:**
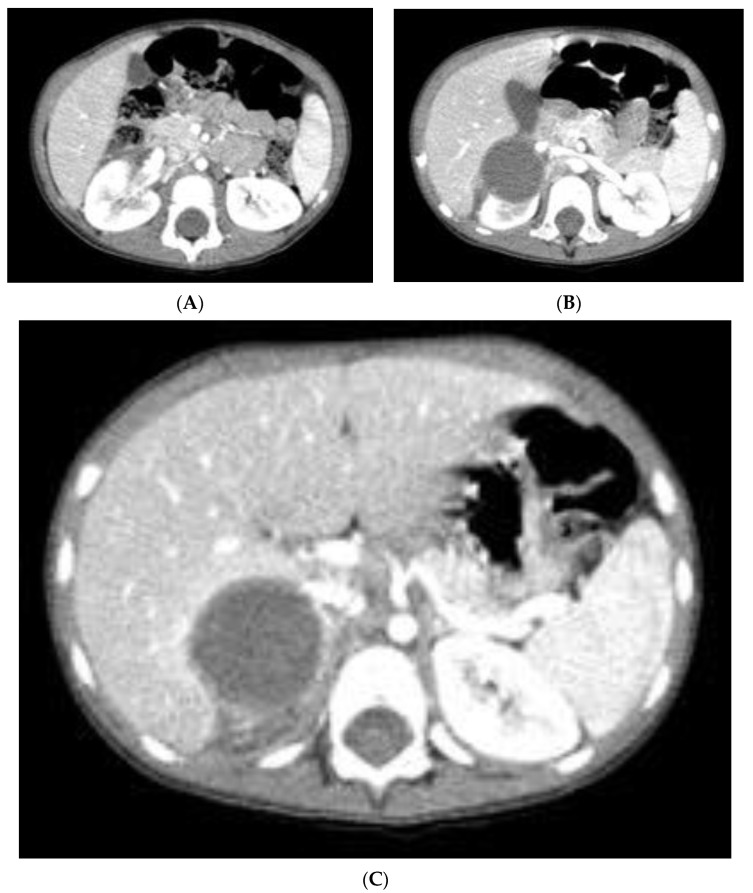
(**A**–**C**). Abdominal CT: (**A**): Transverse scan at the renal hylum, which is not involved by the tumor. (**B**): The mass impresses upon the caudal hepatic lobe without any signs of infiltration. (**C**): The mass indentates the right hepatic lobe, displaying a visible tumor pseudocapsule and a discernible cleavage plane with the liver.

The abdominal CT confirmed an expansive neoformation of 41 mm × 37 mm × 41 mm, with regular margins. It appeared hypodense and minimally uneven, with a density of 35–40 Hounsfield Units (HU). Some lateral septa were observed, and no calcifications were present. The neoformation exerted pressure on the caudal hepatic lobe and displaced the kidney inferiorly, without any signs of infiltration (Figure 2).

**Figure 3:**
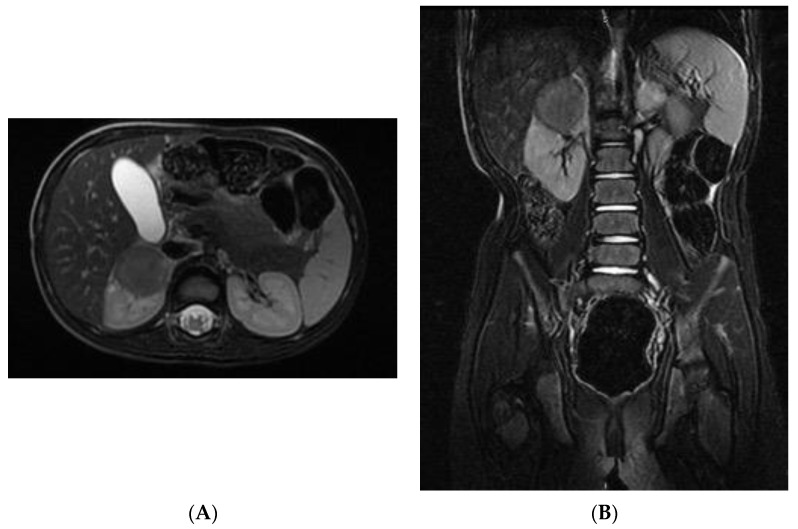
(**A**,**B**). Abdominal MRI: (**A**)—Axial view: the mass occupies the upper renal pole and appears partially exophytic. (**B**)—Coronal view: the mass entirely belongs to the upper renal pole and no lymph node localizations are detectable.

The MRI revealed a hypointense lesion in T1-T2 sequences, displaying no intralesional contrast enhancement in both early and late phases. Only mild contrast enhancement along its margins was observed. Diffusion-weighted imaging (DWI) sequences showed reduced tissue diffusion. In all imaging studies, lymph nodes were found to be uninvolved (Figure 3).

The tumor presented in this case exhibited lower intensity and heterogeneity compared to the typical presentation of WTs. Our radiology team did not univocally provide for a WT diagnosis. The case was discussed at the hospital’s oncology board, and the approved treatment plan involved NSS with a right partial nephrectomy. After an in-depth discussion with the parents regarding the risks and benefits of both MIS and open techniques, as well as the advantages of MIS over the open technique, and the choice between total nephrectomy and NSS, informed consent was obtained for a RAPN.

**Figure 4:**
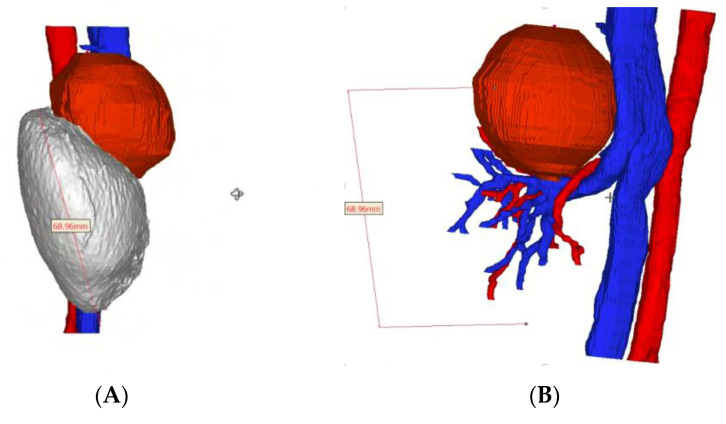

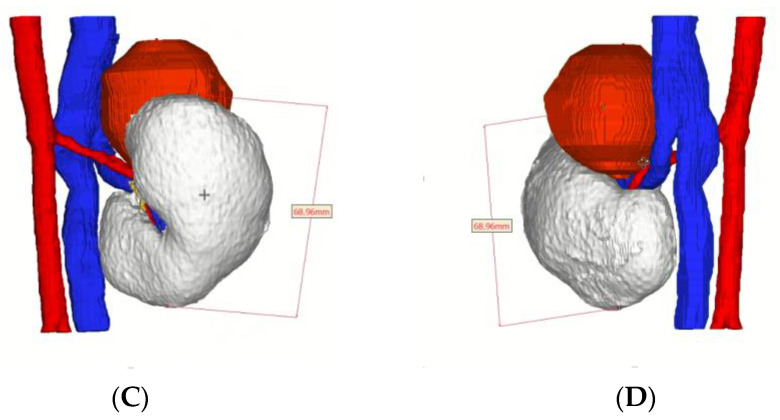
(**A**–**D**). 3D tumor reconstruction: (**A**)—Lateral view showing the tumor occupying the upper pole of the kidney on its anterior surface. (**B**)—After hiding the healthy renal parenchyma, the 3D model displays the superior branch of the renal artery, which feeds the tumor. (**C**)—Posterior view illustrating the relationship between the tumor and main renal artery. (**D**)—Medial view highlighting the relationship between the tumor and the renal vein. Legend: Tumor = red, kidney = white, arterial vessels and aorta = red, venal vessels and inferior vein cava = blue.

A virtual 3D reconstruction of the tumor was obtained (Medics Srl^©^, Moncalieri, Turin, Italy) (Figure 4).

**Figure 5:**
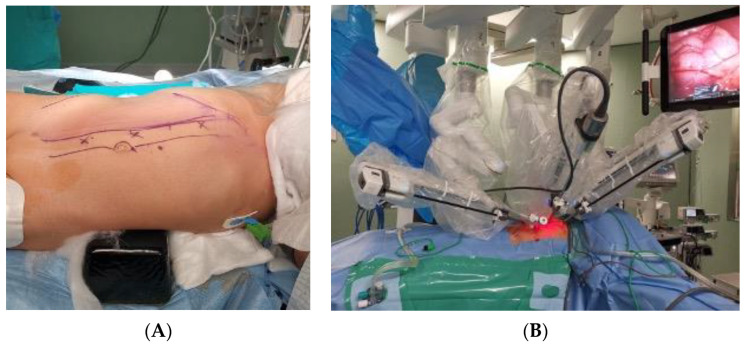
(**A**,**B**). Perioperative assessment. (**A**): Patient positioned on the operating table. Landmarks: midline and pararectal line for trocar placement. (**B**): Da Vinci XI Surgical System is docked.

At surgery, following careful padding and positioning, the patient was inclined at 40° angle on the left flank with slight dorsal hyperextension, in order to increase the distance and resulting operative space between the trocars. The first trocar was inserted using the open technique along the pararectal line. Pneumoperitoneum was established, maintaining pressures at 7 mmHg, in accordance with the European Association of Urology (EAU) Guidelines [15]. Two additional trocars for the robot were placed along the same line, and two 5 mm subxiphoid–umbilical trocars were utilized for the assistant surgeon. The Da Vinci^®^ XI Surgical System was then docked (Figure 5).

**Figure 6:**
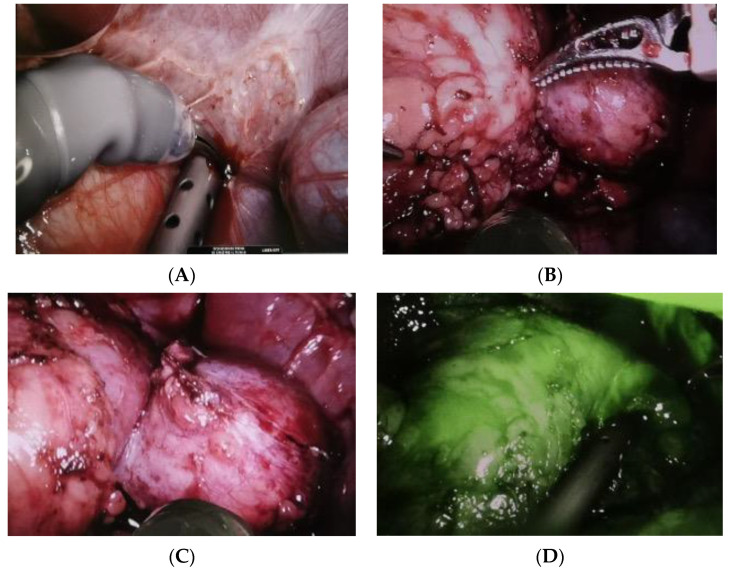
(**A**–**D**). Intraoperative pictures: progressive tumor isolation is achieved. (**A**) The incision starts with the monopolar. (**B**) The tumor is progressively resected. No significant bleeding occurs, thanks to the selective arterial clamping. (**C**) The tumor is completely resected. (**D**) The Indocyanine green test shows good perfusion of the kidney remnant.

After performing the Kocher maneuver to expose the right kidney, the tumor was located and carefully separated from the adrenal gland, which showed no signs of infiltration. Hilar vessels were prepared and secured with vessel loops, but not clamped. Using the superior 3D virtual reconstruction as a guide, only the superior branch of the renal artery supplying the tumor was clamped using a single laparoscopic bulldog clamp (Aesculap, Tuttlingen, Germany). Indocyanine green injection confirmed proper tumor ischemia, with a well-preserved vascularization of the lower two-thirds of the kidney. The partial nephrectomy was executed safely using a monopolar, leaving a margin of healthy renal parenchyma around the tumor (Figure 6).

Hemostasis of the tumor bed was obtained with 5-0 PDS running suture. The cortical was closed with 4-0 vicryl suture and Hem-o-Lock clips^®^ (Teleflex, Research Triangle Park, NC, USA), with perirenal fat intersposed.

The total operative time was 180 min, including the time for robot docking, while console time was 150 min. No warm ischemia time was required, and the estimated blood loss was 20–30 mL. The low body weight did not impede the procedure, which was uneventful, and there was no crashing of instruments.

The post-operative course was uneventful. Early bowel movements began on post-operative day (POD) two, and oral feeding resumed shortly after. The patient was discharged home on the fourth POD due to her constant refusal to mobilize.

Histopathology revealed a WT with central spontaneous necrosis, accounting for 40% of the tissue, and a mixed composition of blastemal (85%) and epithelial (15%) components, without anaplasia. The tumor was completely enclosed by a fibrous tumoral pseudocapsule, with negative surgical margins, classifying it as stage I—intermediate risk.

Following surgery, the patient underwent a 10-cycle adjuvant chemotherapy regimen with vincristine, according to the UMBRELLA SIOP 2016 protocol, regimen 1, for intermediate-risk cases excluding focal anaplasia (post-operative chemotherapy for tumors having primary excision).

One year after surgery, a chest X-ray revealed a suspicious area in the right lung base, which a CT scan determined to be a hilar vessel. Abdominal US displayed normal findings.

As of the date of this work, the follow-up has reached 28 months. The girl remains in good health, with no signs of tumor recurrence.

## 3. Discussion

The standard surgical treatment for unilateral WT remains radical nephrectomy along with lymph node sampling, utilizing a laparotomic transperitoneal approach [16].

According to the UMBRELLA SIOP Protocol, NSS is recommended only in specific cases following neoadjuvant chemotherapy: bilateral, syndromic (for genetic predisposition—less than a year old), nephroblastomatosis [17], high risk of metachronous tumors for or bilateral WT [18], solitary and horseshoe kidney [19], and the constitutional WT1 pathogenic variant that increases the risk of kidney failure [20]. In non-syndromic cases of unilateral WTs, NSS is considered for small tumors (volume <300 mL) without lymph node involvement, where a significant preservation of kidney function is anticipated [21], provided the surgeon can achieve complete tumor excision and prevent tumor spillage [22], as was the case in the scenario described. The conventional open transperitoneal approach is employed for NSS [17], and for the resection of large tumors, it remains the optimal surgical choice [23].

NSS techniques hold particular appeal for use in children. Both radiotherapy and chemotherapy carry nephrotoxic effects, and the risk of developing hypertension, proteinuria, and chronic kidney disease in patients who have survived childhood cancer is not negligible [24]. From this standpoint, neoadjuvant chemotherapy can facilitate NSS and the preservation of renal tissue, inducing a reduction in tumor size and the formation of a pseudocapsule, thereby decreasing the risk of renal rupture during surgery [25].

Historically, laparoscopic treatment has raised concerns due to the absence of tactile feedback, which can increase the risk of tumor rupture due to the frailty of the tumor capsule, and the potential for incomplete staging due to the inadequate abdominal exploration. However, some successful approaches have been reported even prior to chemotherapy [26].

In recent decades, robotic-assisted NSS has become popular in adult urology and has spurred pioneering experiences in pediatric cases. Preoperative imaging plays a crucial role in surgical planning. Based on CT and MRI imaging, three-dimensional (3D) reconstruction enhances the topographic view of the tumor and adjacent structures [27], thereby preventing incidental damage to vessels and surrounding organs.

Previous experiences of RAPN for pediatric WTs are limited, and they are summarized in Table 1. Sala et al. described a case of bilateral WT treated with a right RAPN and a simultaneous left robotic-assisted radical nephrectomy before chemotherapy [14]. To mitigate the risk of tumor rupture and subsequent peritoneal dissemination, Yadav et al. proposed the initial NSS robotic approach for a syndromic WT (WAGR Syndrome) in a child who had undergone prior neoadjuvant chemotherapy [23]. Despite the promising post-operative results, the authors did not provide information on long-term follow-up and recurrence.

Blanc et al. described two syndromic patients who, after receiving neoadjuvant chemotherapy, underwent robotic retroperitoneal partial nephrectomy for WT through a retroperitoneal approach. There was no disease recurrence at 6- and 16-month follow-up. [13]. More recently, Blanc et al. reported their experience with 20 WTs operated on robotically, with no recurrences observed at median follow-up of 2.4 years (range 1.5–3.4), although they did not specify how many of these procedures were NSS [28]. All cases described by Blanc et al. were treated using a retroperitoneal approach for two reasons: first, this approach ensures an untouched peritoneal cavity in case of recurrence or metachronous lesion; second, in the event of positive margins, radiotherapy can be confined to the retroperitoneal space, thus avoiding irradiation of the entire abdomen [13]. In unilateral non-syndromic WTs, NSS should only be considered following neoadjuvant chemotherapy [24].

When the current patient was discussed, several key considerations were factored into the decision-making process.

Firstly, WT typically exhibits MRI heterogeneity and lobules, usually presenting as T1-hyperintense/T2-hyperintense; following intravenous paramagnetic gadolinium administration, it tends to display lower signal intensity compared to the renal cortex [6]. In this particular case, the diagnosis of WT was not immediately evident after the MRI. Despite multiple interdisciplinary meetings, the patient’s care plan could not strictly adhere to the UMBRELLA-SIOP Protocol (neoadjuvant chemotherapy followed by NSS).

Secondly, both MRI and CT imaging indicated a confined and endophytic mass, displaying an unaltered renal anatomy with a low estimated risk of rupture. Given the small size, upper pole location, exophytic nature, and the absence of lymph node involvement, considering NSS was deemed advantageous over radical nephrectomy. Similarly, a biopsy of the mass was not chosen.

Thirdly, the location of the lesion, situated on the anterior aspect of the right kidney, nestled between the inferior vena cava and liver, rendered the robotic approach particularly appealing. This was owing to the enhanced magnification and clearer visualization of the anatomy it offered.

NSS was considered feasible, due to a conjoined adult and pediatric urologic surgical team [29]. While pediatric urologists have extensive experience in robotic surgery for benign conditions [30], the plan for RAPN necessitated collaboration with adult urologists, who routinely perform robotic NSS for renal carcinoma [31]. The decision to use the transperitoneal approach was influenced by the patient’s small size (only 14 kg) and the extensive experience of the adult robotic surgical team with this approach [29].

Ultimately, the 3D reconstruction enabled the preoperative identification of the vessel supplying the tumor and allowed for intraoperative selective arterial clamping, differently from the cases previously described in the literature [12,13,14]. We concur with the view that 3D virtual reconstruction should be regarded as a valuable tool in preoperative assessment, to enable NSS when feasible, thus obviating the need for complete renal ischemia [24,32].

To the best of our knowledge, this is the first reported case of a non-syndromic unilateral WT treated with a transperitoneal RAPN without neoadjuvant chemotherapy and managed surgically with selective arterial clamping. Despite the limited follow-up of 28 months, this approach was successful, resulting in complete excision of the tumor and obviating the need for radiotherapy.

Considering that children with WTs now have a longer life expectancy, and increased risk of long-term sequelae such as hypertension and chronic renal failure is evident, NSS should be the preferred surgical option for WTs when feasible [24]. In this regard, neoadjuvant chemotherapy, 3D preoperative imaging reconstruction, and robotic-assisted surgery in experienced hands all stand as valuable tools that will enhance the feasibility of NSS in pediatric oncology children in the years to come.

**Table 1:** RAPN current literature in pediatric WT management: summary of the published works concerning RAPN for pediatric WT treatment. The last column on the right synthetizes the data of the presented case. Legend: NA = not applicable, ND = not described.

**Author**	Yadav et al. [12]	Blanc et al. [13]	Sala et al. [14]	The presented case
**Year**	2018	2019	2020	NA
**Type**	Case report	Prospective study	Case report	Case report
**Nr. of patients**	1	2	1	1
**Age (yrs)**	1.5	4.7 3.2	3	4
**Surgical approach**	Transperitoneal	Retroperitoneal	Transperitoneal	Transperitoneal
**Arterial clamping**	No	Renal artery	Renal artery	Selective clamping of the superior branch of the renal artery
**Console time**	ND	120′ 110′	90′	150′
**Tumor stage**	I	I	III	I
**Follow-up**	ND	14 13	ND	28
**Medical history**	WAGR Syndrome Right RAPN	Contralateral WT Nephroblastomatosis	Bilateral WT − Right RAPN + contextual left radical nephrectomy	Unilateral non-syndromic WT
**Hospital stay**	ND	4	2	4
**Neoadjuvant chemotherapy**	Yes	Yes	No	No

## 4. Conclusions

RAPN presents a challenging option for managing Wilms tumor. While current experiences are predominantly limited to syndromic WTs, our case suggests a potential role for prechemotherapy RAPN in selected patients with non-syndromic unilateral forms. Additionally, this case implies that the transperitoneal approach can be a viable choice when the tumor is estimated to have a low risk of rupture. The use of 3D virtual reconstruction allows for selective arterial clamping, ensuring intraoperative safety and avoiding complete kidney ischemia.

As current guidelines do not yet define appropriate criteria for combining NSS and MIS in WTs management, study protocols for surgery in pediatric renal oncology are warranted to conduct procedure-related risk assessments and compare them with the traditional surgical strategies currently in use.

## Data Availability

No new data were created. Clinical data are available on request in anonymous form due to privacy restrictions.

## Abbreviations

WT = Wilms tumor, NSS = nephron-sparing surgery, RAPN = robot-assisted partial nephrectomy, CT = computed tomography, MRI = magnetic resonance imaging, DWI = diffusion-weighted images, COG = Children’s Oncology Group, SIOP = International Society of Pediatric Oncology, MIS = minimally invasive surgery, POD = post-operative day, US = ultrasonography, HU = Hounsfield Unit, EAU = European Association of Urology.
